# Supplementary material for: Initial demonstration of a quantum heat engine based on dissipation-engineered superconducting circuits
Source: Nat Commun. 2026 May 5;17:6054. doi: 10.1038/s41467-026-72651-x (PMC13350814; doi:10.1038/s41467-026-72651-x)
Supplement: Supplementary file 1 — Transparent Peer Review file [file 41467_2026_72651_MOESM1_ESM.pdf]

nature portfolio

Peer Review File

Initial demonstration of a quantum heat engine based on dissipation-engineered superconducting circuits

Corresponding Author: Professor Mikko Möttönen

This file contains all reviewer reports in order by version, followed by all author rebuttals in order by version.

Version 0:

Reviewer comments:

Reviewer #1

(Remarks to the Author)
This paper describes an experimental realization of a quantum version of the Otto cycle.
The working medium is a implemented in a superconducting Josephson circuit.
Realizations of quantum heat engines are important in the field of quantum thermodynamics and deserve publication. The experimental Otto engine produces finite power and its efficiency is calculted. The results are close to the theoretical predictions for the Otto cycle.

An essential part of the analysis is missing.
When miniaturizing a heat engine to an individual device fluctuations become important.
The difference between a single shot experiment and the average value become significant.
The experiment has potential to explore this aspect. The full distribution is available.
It can be compared to the TUR.

(Remarks on code availability)

Reviewer #2

(Remarks to the Author)
The manuscript titled, “Initial demonstration of a quantum heat engine based on dissipation-engineered superconducting circuits” by Uusnäkki et al. appears to report the first ever implementation of quantum Otto engine with superconducting circuits. It builds upon extensive expertise and past results with the device referred to as quantum-circuit refrigerator (QCR). QCR is used as thermal bath for a qubit serving as a working medium, to realize the quantum Otto engine.

This work appears to be an important advancement in the field of quantum thermodynamics that needs this kind of concrete experiments. To experimentally validate the performance of quantum thermal machines is of significant interest to the community, and an important step towards both fundamental insights and applications. There seems to have gone in considerable effort to successfully execute this experiment and analyze data to obtain the interesting results of this work. I find the methodology mostly sound with, however, a few concerns that I list down below. I would recommend this manuscript for publication in Nature Communications after the authors have clearly addressed these concerns.

1. Why are the error bars in Fig. 4 (only discernible in Fig. 4b) horizontal in the time axis? I expected it to be along vertical axis for the error margin in population measurements, which I would be interested in.

2. In continuation, it would be good to see how those error bars propagate into markers of Fig. 4c too, but I won’t insist on this and let the authors decide on it.

3. Do all the populations add up to 100% in Fig. 4a and 4b? I am not entirely sure of this, especially at high values of the time duration t. This concerns both the experimental data and the simulation results. If the populations do not add up to 100%,

what is causing the discrepancy?

4. In Fig. 3c, why are the mean IQ of the states represented by markers not at the centers of the identified ellipses? I understand that this has been identified using iterative numerical optimization but I'd not have expected them to deviate so far from the ellipses' centers. Can this be explained?

5. Can you elaborate more quantitatively on how selective is the thermal bath (QCR) in acting upon the qubit transitions? For example, how do the ratios of \Gamma_up/\Gamma_down compare for the different transitions for ge, fe, and higher order terms? It's an important concern how the thermal baths are implemented in quantum thermodynamics, so a short discussion about this selectivity is instructive.

6. In reference to lines 103-105, can you elaborate more on the method how the effective temperature T_eff is "estimated" from approximating to Gibbs state? The definition of Gibbs state specifies strictly the relative populations of the states (Boltzmann's factors), and I think that the populations in Fig. 4 are not necessarily following those Boltzmann ratios throughout the cycles. So do you only consider the states g and e or somehow factor in the populations of higher states?

7. Why is the initial temperature of the qubit in the beginning of the Otto cycles as high as 200 mK in Fig. 4, while base temperature is only 30 mK? This corresponds to an excited population is 25%, which is alarmingly high for a qubit passively thermalized. Typically, the temperature is about 40 - 70 mK in superconducting qubits.

8. It's indicated that this initial state in Fig. 4 is a thermal state before QCR is turned on, whereas in Fig. 1b there is a state "preparation" stage, which leaves me a bit confused. How is it ensured that qubit is in thermal state in Fig. 4? Is it just passive thermalization?

9. I suggest mentioning what is the intrinsic temperature of the qubit, T_int before the QCR activates.

10. Do you have an estimate on the number of thermal photons in the resonator(s)?

Questions out of curiosity regarding the physical mechanism:

- What about the backaction of the heat flows between the qubit (the working medium) and the thermal reservoir on the temperature T_QCR? From earlier works on QCR, I understand that this backaction only changes the electron temperature of the normal metal without affecting T_QCR which is determined by the tunnelling rates that is dominantly dependent on the bias voltage. But when would this break down that T_QCR would be affected by the sheer magnitude of the heat exchanges occurring?

- Only populations have been measured and commented upon in the manuscript. Do you have any insight to what the coherences would be during the Otto cycles? Maybe the simulations could give some clues. In an interesting work, it has been shown that entanglement - a quantum resource - can be generated using pure thermal baths; check out Arxiv: 2401.01776.

Typos to fix:

1. Equation 17 and 18, the order of g and e in the \Gamma ratio need to be corrected.
2. It seems that in many places, Fig. 3 has been mentioned when referring to actually Fig. 4. Please check lines 352, 369 and 395 (I might have missed a few more of these).

(Remarks on code availability)

Version 1:

Reviewer comments:

Reviewer #1

(Remarks to the Author)
The paper describes an experimental realization of a quantum Otto engine based on a superconducting qubit. The current version is satisfactory and can proceed to publication.

(Remarks on code availability)
The paper is a very nice demonstration of the the Otto cycle

Reviewer #2

(Remarks to the Author)
I have gone through the authors' response and the changes they have made. I think they have addressed satisfactorily the issues I raised. I recommend the manuscript for publication in Nature Communications.

(Remarks on code availability)

Open Access This Peer Review File is licensed under a Creative Commons Attribution 4.0 International License, which permits use, sharing, adaptation, distribution and reproduction in any medium or format, as long as you give appropriate credit to the original author(s) and the source, provide a link to the Creative Commons license, and indicate if changes were made.
In cases where reviewers are anonymous, credit should be given to 'Anonymous Referee' and the source.
The images or other third party material in this Peer Review File are included in the article's Creative Commons license, unless indicated otherwise in a credit line to the material. If material is not included in the article's Creative Commons license and your intended use is not permitted by statutory regulation or exceeds the permitted use, you will need to obtain permission directly from the copyright holder.
To view a copy of this license, visit https://creativecommons.org/licenses/by/4.0/

POINT-BY-POINT RESPONSE TO REVIEWER COMMENTS

Reviewer #1:

We thank Reviewer 1 for their comments on the manuscript. Below, we provide our detailed response to all criticism, highlighting the changes made in the manuscript.

Reviewer: This paper describes an experimental realization of a quantum version of the Otto cycle. The working medium is implemented in a superconducting Josephson circuit. Realizations of quantum heat engines are important in the field of quantum thermodynamics and deserve publication. The experimental Otto engine produces finite power and its efficiency is calculated. The results are close to the theoretical predictions for the Otto cycle.

Response: We thank the reviewer for the positive and encouraging feedback on our manuscript, and for recognizing the significance of our experimental realization of a quantum Otto engine within the framework of quantum thermodynamics. We also appreciate the acknowledgement of the relevance of the knowledge gap that this work aims to address, and for the assessment that our results merit publication.

Reviewer: An essential part of the analysis is missing. When miniaturizing a heat engine to an individual device fluctuations become important. The difference between a single shot experiment and the average value become significant. The experiment has potential to explore this aspect. The full distribution is available. It can be compared to the TUR.

Response: We thank the reviewer for pointing out the importance of fluctuations and the connection to thermodynamic uncertainty relations (TURs). Our original manuscript focused on ensemble-averaged thermodynamic quantities of the quantum Otto cycle, obtained from populations reconstructed from a large number of single-shot measurements.

Specifically, each point along the Otto cycle is reconstructed from 10,000 single-shot readout events, from which we infer the level populations using a Gaussian-mixture model. From these populations and the measured eigenfrequencies, we compute the mean internal energy, heat, and work. To characterize fluctuations of the cycle-integrated quantities, we analyze the eight independent repetitions of the first Otto cycle, and we find that the relative fluctuations (standard deviation divided by mean) are relatively small, indicating that in our parameter regime the engine operates close to its mean-field behavior.

From the viewpoint of stochastic thermodynamics, a quantitative comparison with TURs requires defining trajectory-level heat currents and the corresponding entropy production. While our experiment already provides access to ensemble fluctuations of the cycle output, a complete TUR analysis for this multi-level and time-dependent open quantum system with a temperature-tunable reservoir would require additional modelling and a measurement setup tailored to trajectory-level statistics.

Note that in the current experimental scheme, we cannot deduce the transmitted heat and work for single trajectories because we first prepare the qubit in its initial state, evolve the qubit for a variable amount of time, read out the state of the qubit in a single shot, and repeat this preparation-evolution-readout cycle for 10,000 times. Thus, we do not monitor the qubit continuously. We do this to avoid any mid-cycle measurements and the corresponding effect of the quantum backaction of the readout on the measured ensemble-averaged quantities. Such backaction is drastically large for single-shot measurements, but smaller for weak continuous measurements, which however, we have not implemented in the laboratory to date. Note that the situation is distinctively different for classical heat engines that are typically less perturbed by the measurement apparatus.

Because of the above-mentioned reasons, we have limited ourselves to quantifying the experimentally observed fluctuations of work and heat and discussing their implications. We have revised the manuscript and added a discussion on this matter in lines **431–448** and identified a detailed TUR test as a promising direction for future work.

POINT-BY-POINT RESPONSE TO REVIEWER COMMENTS

Reviewer #2:

We thank Reviewer 2 for their insightful and helpful comments. Below, we provide our detailed response to all criticism, highlighting the changes made in the manuscript.

Reviewer: The manuscript titled, "Initial demonstration of a quantum heat engine based on dissipation-engineered superconducting circuits" by Uusnäkki et al. appears to report the first ever implementation of quantum Otto engine with superconducting circuits. It builds upon extensive expertise and past results with the device referred to as quantum-circuit refrigerator (QCR). QCR is used as thermal bath for a qubit serving as a working medium, to realize the quantum Otto engine.

This work appears to be an important advancement in the field of quantum thermodynamics that needs this kind of concrete experiments. To experimentally validate the performance of quantum thermal machines is of significant interest to the community, and an important step towards both fundamental insights and applications. There seems to have gone in considerable effort to successfully execute this experiment and analyze data to obtain the interesting results of this work. I find the methodology mostly sound with, however, a few concerns that I list down below. I would recommend this manuscript for publication in Nature Communications after the authors have clearly addressed these concerns.

Response: We thank the reviewer for the clear and detailed summary of our work, and we are grateful for their interest in the experiments and appreciation of the effort and scientific work involved. We are pleased that the reviewer regards our results as an important advancement in experimental quantum thermodynamics, and we appreciate the recommendation for publication in Nature Communications, provided that the concerns raised are clearly addressed. We respond to each of the reviewer's comments point by point below.

Reviewer: 1. Why are the error bars in Fig. 4 (only discernible in Fig. 4b) horizontal in the time axis? I expected it to be along vertical axis for the error margin in population measurements, which I would be interested in.

Response: We thank the reviewer for pointing out the issue with the visibility in the error bars. The error bars were plotted in a vertical setting with |--o--| shaped error bars, but as the error bars are very small in the case of the repetitions in Fig. 4b, they appear horizontal. We apologize for this confusion, and therefore, we have revised the error bars for the main results. We have also added error bars to Fig. 4a and 4b as well by calculating the error in the populations from the GMM model using a binomial approximation where $\sigma_m = \sqrt{p_m(1-p_m)/N_{\text{tot}}}$ for state m.

Reviewer: 2. In continuation, it would be good to see how those error bars propagate into markers of Fig. 4c too, but I won't insist on this and let the authors decide on it.

Response: We agree with the reviewer that it would be insightful to have the error bars propagate also to Fig. 4c, and we have added them to the revised manuscript. The uncertainty of the mean energy is calculated using standard error propagation for the mean energy equation $\mathrm{E} = \sum_m \hbar\omega_m p_m$ assuming that the error in the eigenfrequency is small. For the temperature, we use Monte Carlo sampling to find an estimate for the temperature error. The temperature is found by maximizing the fidelity between the measured populations and thermal state populations for a given temperature. To gain an error estimate, we repeat this maximization by sampling population data for *n* = 1000 samples from a multinomial distribution determined by the measured populations and the total count, and then we take the mean and standard deviation from the acquired temperature data. This is repeated for each measurement point. To highlight this, we added a note to lines **930–937** explaining this method for the error.

Reviewer: 3. Do all the populations add up to 100% in Fig. 4a and 4b? I am not entirely sure of this, especially at high values of the time duration t. This concerns both the experimental data and the simulation results. If the populations do not add up to 100%, what is causing the discrepancy?

Response: We agree with the reviewer that it is not obvious from Figs. 4a and 4b whether the populations add up to 100%. As stated in the manuscript, the populations are obtained from calculating the amount of measured single shot data points inside each ellipse corresponding to each state and then normalizing them with the total amount of points in all ellipses. This is explained in more detail in the Methods section (Transmon readout and state identification, Eq. 29). This ensures that the populations add up to 100% at all time steps.

Reviewer: 4. In Fig. 3c, why are the mean IQ of the states represented by markers not at the centers of the identified ellipses? I understand that this has been identified using iterative numerical optimization but I'd not have expected them to deviate so far from the ellipses' centers. Can this be explained?

Response: The mean IQ states represented by the markers are the mean values of the three 'raw' calibration data sets from the attempted but imperfect initial states $|\mathrm{g}\rangle$, $|\mathrm{e}\rangle$, and $|\mathrm{f}\rangle$, whereas the centers of the ellipses denote the mean values of the fitted Gaussian distributions for the corresponding ideal states. The difference arises from the number of distributions in the fitting as we fit for four distributions, the first three states and the rest of the high-lying states in the fourth distribution. As the prepared states have some leakage to higher (and lower) states, the data set means differ from the distribution means, but still lie inside the ellipses depicting the validity of the Gaussian fit and the spread of the data. To make this clearer, we added a small note in the end of the Figure 3 caption.

Reviewer: 5. Can you elaborate more quantitatively on how selective is the thermal bath (QCR) in acting upon the qubit transitions? For example, how do the ratios of \Gamma_up/\Gamma_down compare for the different transitions for ge, fe, and higher order terms? It's an important concern how the thermal baths are implemented in quantum thermodynamics, so a short discussion about this selectivity is instructive.

Response: The QCR aims to unconditionally cool and heat the coupled system since the photon-assisted tunnelling allows all frequencies ω_q for which $\Delta - \hbar\omega_q < e|V_{\mathrm{QCR}}| < \Delta$ for cooling and $e|V_{\mathrm{QCR}}| > \Delta + \hbar\omega_q$ for heating. However, there is some inherent selectivity arising from this conditions which depends on the used QCR bias voltage. We can use Eq. (16) and approximate the ratios of thermal emission and absorption using the eigenfrequencies and voltages used in the Otto cycles depicted in Fig 1b in the manuscript. We display the results below in Figure 1. The Γ_{nm}/Γ_{mn} ratios in the first subplot 1a show that the ratios are all within a 5 % deviation from one another in the heating stage of the cycle, but the deviation is somewhat higher in the cooling stage where the ratio of relaxation and excitation rate is larger for the ground state compared to higher states . However, if we calculate the effective QCR temperature associated with each transition $T_{\mathrm{QCR}}^{mn} = \frac{\hbar\omega_{mn}}{k_{\mathrm{B}}}\left[\ln\left(\frac{\Gamma_{\mathrm{QCR}}^{nm}}{\Gamma_{\mathrm{QCR}}^{mn}}\right)\right]^{-1}$, we see that the change in the eigenfrequency compensates for this difference in transition rates and the effective temperatures are essentially the same for each transition. This shows that the QCR induced transition rates are not very selective on the different transitions, and the QCR appears as a thermal bath to the coupled qubit where the transitions satisfy the detailed balance equation given in Eq. 18. We added a short discussion on this selectivity in the manuscript to lines **742-745**.

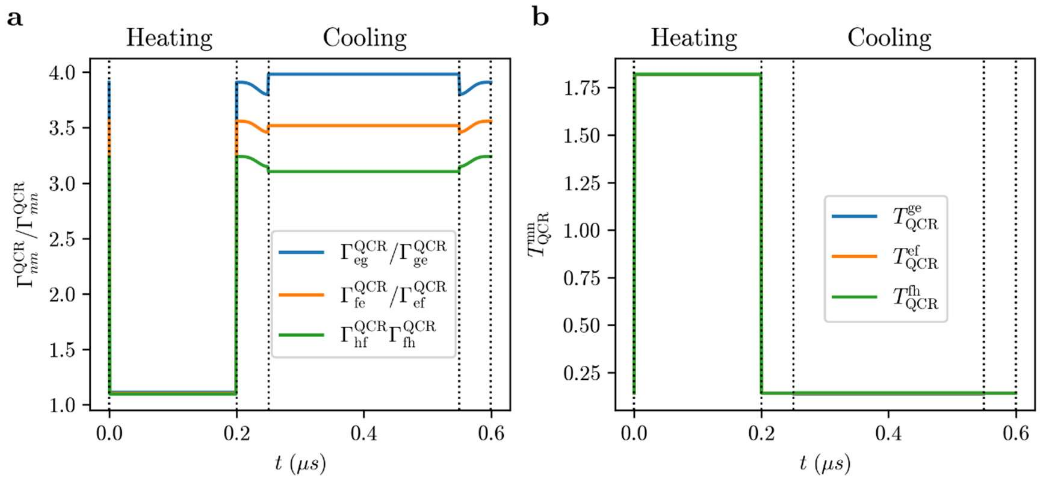

Figure 1. (**a**) Relaxation over excitation rate of QCR-induced tunnelling rates as a function of time for an Otto cycle. (**b**) Effective temperature of the QCR from the given transition rates as a function of time for an Otto cycle.

Reviewer: 6. In reference to lines 103-105, can you elaborate more on the method how the effective temperature T_eff is “estimated” from approximating to Gibbs state? The definition of Gibbs state specifies strictly the relative populations of the states (Boltzmann’s factors), and I think that the populations in Fig. 4 are not necessarily following those Boltzmann ratios throughout the cycles. So do you only consider the states g and e or somehow factor in the populations of higher states?

Response: The effective temperature T_{eff} is estimated by finding the closest thermal state to the measured populations. This is done by maximizing the fidelity function $F(T_{\mathrm{eff}}) = \left(\sum_k \sqrt{p_k^{\mathrm{th}}(T_{\mathrm{eff}})p_k}\right)^2$. This yields the temperature of the closest thermal state. The states k

considered here are the states $|g\rangle, |e\rangle, |f\rangle$ and $|h, i, j\rangle$ captured by the GMM model. This is highlighted in lines **922–929**.

Reviewer: 7. Why is the initial temperature of the qubit in the beginning of the Otto cycles as high as 200 mK in Fig. 4, while base temperature is only 30 mK? This corresponds to an excited population is 25%, which is alarmingly high for a qubit passively thermalized. Typically, the temperature is about 40 - 70 mK in superconducting qubits.

Response: The initial temperature (with zero QCR bias) obtained from the nearest thermal state distribution is 160 mK which is much higher than the base temperature. The given base temperature in the manuscript was 30 mK, but this is a typo and the actual temperature is around 40 mK, fixed now in the revision. The base temperature is the temperature of the dilution refrigerator mixing chamber flange with which the sample holder is in thermal contact. However, the effective temperature that the qubit experiences is increased by other factors such as thermal noise arising from multiple sources including the readout and auxiliary resonators, flux and qubit drive lines, as well as passively from the QCR junction. It has been recorded also previously that the effective temperature of the qubit in a QCR-Transmon setup is higher than the base temperature due to these couplings [T. Mörstedt *et al*. Phys. Rev. Research 7, L042010 (2025), T. Wallace *et al*. Sci. Rep. 14, 13755 (2024)]. Fortunately, such increased qubit temperature does not hinder our observation of the Otto cycle.

Reviewer: 8. It's indicated that this initial state in Fig. 4 is a thermal state before QCR is turned on, whereas in Fig. 1b there is a state "preparation" stage, which leaves me a bit confused. How is it ensured that qubit is in thermal state in Fig. 4? Is it just passive thermalization?

Response: The state of the qubit is passively thermalized to its environment, and this is the state at which we start the process. The preparation stage in Fig. 1b refers to the fact that we label the starting point of the Otto cycle to be at the higher temperature stage as indicated by the point 'A' of the cycle in Fig. 1a. The idea of the preparation stage is that one can control the length of the initial heating pulse in such a way, that we could in the future prepare/move the state of the qubit from the passively thermalized state to a high enough thermal state that the cycle would start and end at equal temperatures. However, we could not carry out these measurements as the sample had degraded.

Reviewer: 9. I suggest mentioning what is the intrinsic temperature of the qubit, T_int before the QCR activates.

Response: We agree that the intrinsic temperature should be mentioned and that is added on line **356** in the manuscript.

Reviewer: 10. Do you have an estimate on the number of thermal photons in the resonator(s)?

Response: We estimate the number of thermal photons in the 40 mK base temperature in the readout resonator to be 1.3e-4 and in the auxiliary resonator to be 3.7e-3 given their respective resonance frequencies 7.436 GHz and 4.670 GHz. However, these numbers may be higher in practice owing to parasitic noise.

Reviewer: Questions out of curiosity regarding the physical mechanism:

- What about the backaction of the heat flows between the qubit (the working medium) and the thermal reservoir on the temperature T_QCR? From earlier works on QCR, I understand that this backaction only changes the electron temperature of the normal metal without affecting T_QCR which is determined by the tunnelling rates that is dominantly dependent on the bias voltage. But when would this break down that T_QCR would be affected by the sheer magnitude of the heat exchanges occurring?

Response: There are multiple back-action channels between the qubit and the electron temperature of the normal metal in the QCR. The QCR temperature is defined via the tunnelling rates as mentioned, but these tunnelling rates are also functions of the normal metal temperature T_{N}. For bias voltages in the cooling regime, the normal metal temperature significantly affects the effective temperature that the qubit experiences as it approaches the ideal $T_{\mathrm{QCR}} = T_{\mathrm{N}}/2$ regime [M. Silveri *et al*. Phys. Rev. B 96, 094524 (2017)]. However, for high bias voltages in the hot-environment case, this sensitivity vanishes and the effective temperature and the tunnelling rates are mainly determined by the bias voltage. Thus investigating the backaction of the qubit on the QCR is only important in the cooling regime. Fortunately, the heat flow from the qubit to the QCR electron cloud is very low and consequently has only a miniscule effect on T_{N} and hence on T_{QCR}. We can approximate this by calculating the amount of power released to the QCR during the cooling stroke from the data in Fig. 4 in the manuscript and compare it to how much the temperature needs to change to match this power given the electron-phonon thermal conductance for the normal metal $G = 5\Omega_{\mathrm{N}}\Sigma_{\mathrm{Cu}}T_{\mathrm{N}}^4$. This analysis yields a change in T_{N} well below one millikelvin, which is insignificant for the experiment.

Reviewer: - Only populations have been measured and commented upon in the manuscript. Do you have any insight to what the coherences would be during the Otto cycles? Maybe the simulations could give some clues. In an interesting work, it has been shown that entanglement - a quantum resource - can be generated using pure thermal baths; check out Arxiv: 2401.01776.

Response: In this work, we only consider thermal states for the initial demonstration of the cycle since these are the “natural” states of the qubit and since the QCR essentially drives the qubit to a thermal state dependent on the bias voltage. Thus, we also used thermal states in the simulations to which the measured values are compared to. However, we can examine the coherences of the transmon in the simulations if we compare the initial state to some other state than thermal state with off-diagonal matrix elements and calculate the von Neumann entropy and off-diagonal norm to display how the coherences behave. From Figure 2 below we observe that during the Otto cycles for different initial states with off-diagonal elements, the states approach a thermal state within the first heating pulse and the von Neumann entropies start from low and then reach around the same level as the thermal state. For the coherent state with higher populations for the higher states, the system reaches a thermal state with higher temperature and higher von Neumann entropy, which represents the fact that a higher-temperature thermal state is more mixed. We can also observe that the off-diagonal elements diminish over time as the norm approaches zero. It seems that the cycle forces the state to a thermal state quite quickly during the first stroke of the cycle.

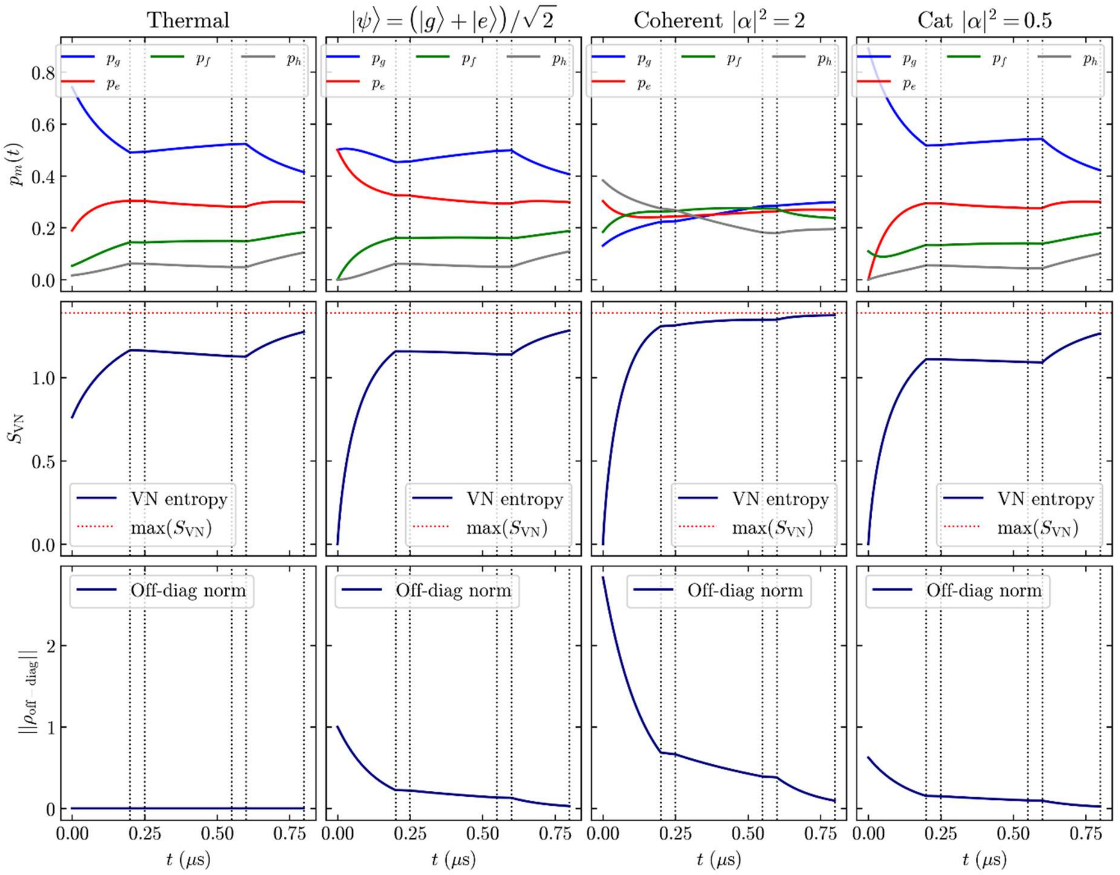

Figure 2. Simulated transmon populations (first row), von Neumann entropy (second row), and off-diagonal norm of the density matrix (third row) for four different initial states during the first three Otto cycles. The used initial states from left to right columns are a thermal state, a superposition of the ground and first excited state, a coherent state, and a cat state.

Reviewer: Typos to fix:

1. Equation 17 and 18, the order of g and e in the \Gamma ratio need to be corrected.
2. It seems that in many places, Fig. 3 has been mentioned when referring to actually Fig. 4. Please check lines 352, 369 and 395 (I might have missed a few more of these).

Response: We thank the reviewer for pointing out these typos in the manuscript. The mistakes are fixed and highlighted in the revised manuscript.
